# The therapeutic and biomarker significance of ferroptosis in chronic myeloid leukemia

**DOI:** 10.3389/fimmu.2024.1402669

**Published:** 2024-07-04

**Authors:** Fangmin Zhong, Xueru Zhang, Zihao Wang, Xiaolin Li, Bo Huang, Guangyao Kong, Xiaozhong Wang

**Affiliations:** ^1^ Jiangxi Province Key Laboratory of Immunology and Inflammation, Jiangxi Provincial Clinical Research Center for Laboratory Medicine, Department of Clinical Laboratory, The Second Affiliated Hospital, Jiangxi Medical College, Nanchang University, Nanchang, Jiangxi, China; ^2^ National and Local Joint Engineering Research Center of Biodiagnosis and Biotherapy, The Second Affiliated Hospital of Xi’an Jiaotong University, Xi’an, Shaanxi, China

**Keywords:** chronic myeloid leukemia, ferroptosis, immune microenvironment, treatment, machine learning, diagnosis

## Abstract

**Background:**

The relationship between ferroptosis and the progression and treatment of hematological tumors has been extensively studied, although its precise association with chronic myeloid leukemia (CML) remains uncertain.

**Methods:**

Multi-transcriptome sequencing data were utilized to analyze the ferroptosis level of CML samples and its correlation with the tumor microenvironment, disease progression, and treatment response. Machine learning algorithms were employed to identify diagnostic ferroptosis-related genes (FRGs). The consensus clustering algorithm was applied to identify ferroptosis-related molecular subtypes. Clinical samples were collected for sequencing to validate the results obtained from bioinformatics analysis. Cell experiments were conducted to investigate the therapeutic efficacy of induced ferroptosis in drug-resistant CML.

**Results:**

Ferroptosis scores were significantly lower in samples from patients with CML compared to normal samples, and these scores further decreased with disease progression and non-response to treatment. Most FRGs were downregulated in CML samples. A high ferroptosis score was also associated with greater immunosuppression and increased activity of metabolic pathways. Through support vector machine recursive feature elimination (SVM-RFE), least absolute shrinkage selection operator (LASSO), and random forest (RF) algorithms, we identified five FRGs (ACSL6, SLC11A2, HMOX1, SLC38A1, AKR1C3) that have high diagnostic value. The clinical diagnostic value of these five FRGs and their effectiveness in differentiating CML from other hematological malignancies were validated using additional validation cohorts and our real-world cohort. There are significant differences in immune landscape, chemosensitivity, and immunotherapy responsiveness between the two ferroptosis-related molecular subtypes. By conducting cellular experiments, we confirmed that CML-resistant cells are more sensitive to induction of ferroptosis and can enhance the sensitivity of imatinib treatment.

**Conclusion:**

Our study unveils the molecular signature of ferroptosis in samples from patients with CML. FRG identified by a variety of machine learning algorithms has reliable clinical diagnostic value. Furthermore, the characterization of different ferroptosis-related molecular subtypes provides valuable insights into individual patient characteristics and can guide clinical treatment strategies. Targeting and inducing ferroptosis holds great promise as a therapeutic approach for drug-resistant CML.

## Introduction

Chronic myeloid leukemia (CML) is a hematological neoplasm initiated by the fusion gene BCR-ABL (1). The introduction of tyrosine kinase inhibitors (TKIs), such as imatinib, has significantly enhanced therapeutic efficacy for CML patients while substantially improving their prognosis (2). However, intricate escape mechanisms employed by tumor cells inevitably hinder the effectiveness of these kinase drugs and lead to the gradual development of drug resistance in patients with CML (3). These resistance mechanisms include both primary and secondary factors; among them, mutations in BCR-ABL protein play a crucial role (4). Despite the advancements achieved in the development of novel TKIs that target specific mutation sites associated with enhanced treatment response in CML (5); challenges persist due to emerging new mutation sites over time as well as non-mutation-based resistance mechanisms that arise during therapy course (6). Therefore, a more comprehensive analysis of the molecular biology and metabolic characteristics of CML cells holds significant clinical value for treatment decision-making and prognosis evaluation in patients with CML.

Ferroptosis is a novel form of cell death, characterized by distinct mechanisms and morphology compared to apoptosis, necrosis, and autophagy (7). The process is initiated by intracellular divalent iron or ester oxygenase, resulting in the peroxidation of highly expressed unsaturated fatty acids on the cell membrane and subsequent induction of ferroptosis (8–10). Morphological changes observed in cells undergoing ferroptosis include disruption of the cell membrane, mitochondrial outer membrane, and loss of cristae (11). The occurrence of ferroptosis involves various regulatory pathways such as the classical GPX4-regulated mechanism (Cyst(e)ine/GSH/GPX4 axis) (12), as well as GPX4-independent mechanisms like NAD(P)H/FSP1/CoQ10 axis (13), GCH1/BH4/DHFR axis (14), and squalene accumulation. Additionally, signaling pathways including E-cadherin-NF2-Hippo-YAP, AMPK, and HIF2α-HILPDA also modulate cellular sensitivity to ferroptosis (15–17). Numerous studies have demonstrated that targeted induction of ferroptosis holds promise as a new therapeutic strategy for acute myeloid leukemia (18–20). Liu et al.’s research revealed TXNRD1’s crucial role in cysteine depletion-induced ferroptosis in CML cells *in vitro (* 18, 21). However, there remains a limited understanding regarding the relationship between ferroptosis and CML, as well as its underlying mechanism, necessitating further comprehensive investigation.

In this study, we conducted a comprehensive analysis of the ferroptosis pathway and gene expression characteristics in CML, aiming to elucidate the underlying mechanism of ferroptosis and its interaction with the CML tumor microenvironment. Through multi-group cohort analysis, we validated the diagnostic value of ferroptosis-related genes (FRGs) in CML, and subsequent experiments further confirmed the potential therapeutic significance of targeting ferroptosis in overcoming drug resistance.

## Methods

### Data acquisition and preprocessing

The sequencing data of CML cohorts GSE13159, GSE144119, GSE4170, and GSE44589 were obtained from the Gene Expression Omnibus (GEO) database. The analysis cohort for this project was the GSE13159 cohort, which consisted of 76 CML samples and 74 normal samples. Raw sequencing data were downloaded and normalized for subsequent analysis. The validation cohort (GSE144119) included 48 newly diagnosed CML samples, 32 remission CML samples, and 17 normal samples that were converted to transcripts per kilobase million (TPM) values. For clinical validation purposes, transcriptome sequencing was performed on five chronic-phase CML samples, five blast crisis samples, and five normal control samples with written consent from patients approved by the Ethics Committee of the Second Affiliated Hospital of Nanchang University; these data were also transformed into TPM values for further validation. To differentiate between other types of leukemia such as acute lymphoblastic leukemia (750 cases), acute myeloid leukemia (542 cases), chronic lymphocytic leukemia (448 cases), and myelodysplastic syndromes (206 cases), a subset of the GSE13159 cohort was utilized. Furthermore, we used the imatinib-treated sample dataset from GSE44589 containing 198 sequenced samples to evaluate treatment response in CML patients. Additionally, single-cell RNA-seq data from the GES76312 cohort were employed to visualize clusters using the uniform manifold approximation and projection (UMAP) algorithm. Finally, we retrieved ferroptosis pathway genes from the MSigDB database (https://www.gsea-msigdb.org/gsea/msigdb/index.jsp).

### Differential expression analysis of FRG

The “limma” software package was employed for conducting differential expression analysis of FRG. Adjusted p values below 0.05 were considered significant, indicating the presence of differentially expressed FRGs (DEFRGs) between CML and normal samples. Subsequently, we performed Gene Ontology (GO) annotation and Kyoto Encyclopedia of Genes and Genomes (KEGG) pathway enrichment analysis on these genes using the “clusterProfiler” package (22). To quantify the activity of a biological pathway or gene set, we utilized the Gene Set Variation Analysis (GSVA) algorithm to calculate an enrichment score (23).

### Correlation analysis and protein-protein interaction (PPI) network construction

The Spearman method was employed for correlation analysis. The STRING database (https://string-db.org/) was used to analyze the PPI of DEFRG. Subsequently, the PPI network was visualized using Cytoscape software.

### Analysis of immune cell infiltration

The estimation of immune cell infiltration was conducted by employing the deconvolution algorithm “CIBERSORT” to accurately quantify the proportions of 22 distinct immune cell types based on the gene expression profiles of individual samples (24).

### Potential regulatory mechanisms associated with ferroptosis

Weighted correlation network analysis (WGCNA) was employed to identify the genes associated with ferroptosis scores in the GSE13159 cohort (25). Pearson correlation analysis was utilized to construct the adjacency matrix for all matched genes, and the scale-free topology of this matrix was established based on an optimal soft threshold power. Subsequently, the adjacency matrix was transformed into a topological overlap matrix (TOM). By employing the TOM dissimilarity measure, modules consisting of genes exhibiting similar expression patterns were identified through average linkage hierarchical clustering, with a minimum module size set at 30 and a cut height at 0.2. Finally, an evaluation of the correlation between module signature genes (MEs) and ferroptosis score was performed.

### Analysis of the diagnostic value of FRGs

To identify diagnostic biomarkers for CML, three machine learning algorithms, namely support vector machine recursive feature elimination (SVM-RFE), least absolute shrinkage selection operator (LASSO), and random forest (RF) were employed to screen the diagnostic FRGs. Additionally, LASSO regression analysis was used to calculate regression coefficients for the diagnostic FRGs, and a CML risk score diagnostic model was constructed using the following formula:


$$Risk\;score=\sum_{1}^{i}\left(Coefi\;*\;ExpGenei\right),\;$$

where i represents the specific diagnostic FRG and “Coef” and “ExpGene” denote the regression coefficient and expression value of that particular FRG respectively. By constructing this risk score model, we can further assess the combined diagnostic value of FRGs.

### Revealing molecular subtypes via FRG expression profiling

To comprehensively assess inter-individual variations in CML patients, we employed the “ConsensusClusterplus” package to conduct a cluster analysis of CML samples based on the expression profiles of the diagnostic FRGs, aiming to identify distinct molecular subtypes within CML (26). The robustness and stability of the clustering results were confirmed through 1000 iterations. Additionally, principal component analysis (PCA) was utilized for classification validation.

### Prediction of the sensitivity of CML samples to TKI treatment and immunotherapy

The expression matrix and drug response data of blood cell lines from the Cancer Genome Project (CGP) database were utilized in this study to predict the half-maximal inhibitory concentrations (IC50) of CML samples to TKIs. This prediction was made using the “pRRophetic” package, a computational tool commonly used for such analyses (27). To further investigate the response of different risk score groups towards anti-PD-1 and anti-CTLA4 immune checkpoint inhibitors, we employed the “SubMap” algorithm available at a publicly accessible website called GenePattern. The SubMap algorithm is widely recognized for its ability to forecast treatment responses based on gene expression profiles. To assess the level of immune escape exhibited by tumor cells in CML samples, we computed the TIDE score using an established online resource known as Tumor Immune Dysfunction and Exclusion (TIDE).

### Construction of microRNA (miRNA) regulatory network for diagnostic FRGs

We employed miRTarBase, miRDB, and TargetScan databases to predict the binding sites of miRNAs on CML diagnostic ARGs. Subsequently, we filtered out the miRNA-target pairs that were predicted by all three databases. The GSE90773 cohort was utilized to identify differentially expressed miRNAs between CML cells and normal cells, which served as the basis for constructing the miRNA regulatory network.

### *In vitro* experiments

The CML cell line K562 was cultured in RPMI1640 medium supplemented with 10% fetal bovine serum and 1% penicillin-streptomycin in a humidified incubator saturated with 5% CO2 at 37°C. The K562 cells were exposed to imatinib, and the concentration was gradually increased until the development of K562/IR cells capable of sustained growth in a medium containing 1μM of imatinib. This concentration is considered physiologically relevant and may simulate the peak plasma/serum level of imatinib (5μM). Transcriptome sequencing analysis was conducted on K562, K562/IR, K562/IR control, and erastin-treated K562/IR cells. The processing procedure employed in this study was based on our previous research (28). The concentration of imatinib was gradually increased until the induction of resistant cells was completed. Cell viability was assessed using the cell counting kit-8 (CCK-8) assay. For this assay, 5-e3 cells were seeded in 96-well plates, and each group was repeated three times. After the indicated culture time, 10 μL of CCK8 solution was added, followed by incubation at 37°C for 2 hours. The optical density (OD) value at 450 nm was measured using a microplate reader. Apoptosis detection involved staining cells with the Annexin V-PE/7-AAD apoptosis detection kit and subsequent examination in a flow cytometer. Additionally, reactive oxygen species (ROS) were detected using a fluorescent probe DCFH-DA in flow cytometry. The levels of GSH and GSSH were determined using Solarbio’s BC1175 and BC1185 kits, respectively. Bioss’ AK091 kit was used for GPX4 activity measurement. All reagents were employed following the manufacturer’s instructions. Cell homogenization was performed using lysate buffer to facilitate the reaction between REDOX substances in the sample and reagents, resulting in the formation of adducts that can be quantified through colorimetry.

### Statistical analysis

All analyses were conducted using the R software and corresponding software packages. Differences between two or more groups were assessed using the Wilcoxon rank sum test and the Kruskal-Wallis test, respectively. The diagnostic value of biomarkers was determined through receiver operating characteristic (ROC) curve analysis. A bilateral P-value less than 0.05 indicates a statistically significant difference.

## Results

### Molecular characteristics linked to ferroptosis in CML

**Figure 1:**
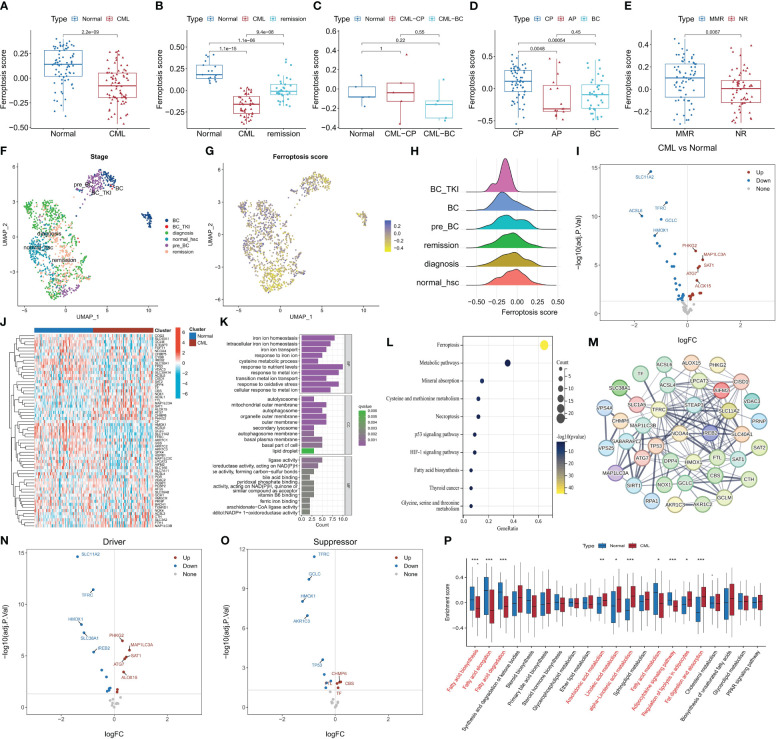
The characteristics of ferroptosis score and FRG expression in CML samples. **(A-E)** Differences in ferroptosis scores between CML samples and normal samples were observed in various datasets: **(A)** GSE13159, **(B)** GSE144119, **(C)** our clinical cohort, **(D)** GSE4170, **(E)** GSE44589. **(F-H)** UMAP analysis of the CML single-cell sequencing dataset GSE76312 revealed the distribution of ferroptosis scores among different patients. **(I-J)** Volcano map **(I)** and heat map **(J)** illustrated the expression characteristics of FRG. **(K, L)** Functional annotation **(K)** and pathway enrichment analysis **(L)** were conducted on DEFRG. **(M)** PPI network analysis was performed on DEFRG. **(N, O)** Expression characteristics of ferroptosis suppressors and drivers were examined. **(P)** Differences in lipid metabolic pathway scores between normal and CML samples. BC refers to blast crisis, CP to chronic phase, MMR to major molecular response, and NR to no response. *p < 0.05; **p < 0.01; ***p < 0.001.

We conducted a comprehensive evaluation of ferroptosis activity and molecular characteristics in CML using transcriptomics analysis. The GSVA algorithm was utilized to calculate ferroptosis scores, revealing significantly lower ferroptosis scores in CML samples compared to normal samples (**Figure 1A**), while the ferroptosis score increased following treatment remission (**Figure 1B**). Patients in blast crisis (BC) exhibited even lower ferroptosis scores than those in the chronic phase (CP) (**Figures 1C, D**) (Due to the limited sample size and vulnerability to individual outliers, although **Figure 1C** does not exhibit a statistically significant difference, the overall trend persists that BC patients display lower ferroptosis scores compared to CP patients.), and individuals with major molecular responses displayed higher ferroptosis scores compared to non-responders (**Figure 1E**). Single-cell analysis consistently demonstrated a trend of decreased ferroptosis scores in CML patients, particularly those in BC, which subsequently increased after treatment with TKI (**Figures 1F-H**). Differential expression analysis indicated the down-regulation of numerous genes associated with ferroptosis in CML samples (**Figures 1I, J**), including those involved in iron ion homeostasis, mitochondrial outer membrane function, and ligase activity (**Figure 1K**). These differentially expressed genes were primarily enriched in signaling pathways related to ferroptosis, metabolic pathways, mineral absorption, and cysteine and methionine metabolism (**Figure 1L**). PPI network analysis identified STEAP3, TFRC NCQA4 TP53 IREB2 as hub genes within the network formed by these DEFRG (**Figure 1M**). Volcano plot analysis further revealed down-regulation of gene expression for various suppressors of ferroptosis in CML samples (**Figures 1N, O**). Therefore, we speculate that the observed lower ferroptosis score in CML may be attributed to an overall decrease in inhibition of this process within cancer cells indicating their heightened susceptibility towards undergoing cell death through the mechanism of the ferroptosis pathway. Ferroptosis is closely linked to lipid metabolism, and our findings reveal a significant increase in the activity of unsaturated fatty acids such as linoleic acid, arachidonic acid, and α-linolenic acid in CML (**Figure 1P**). Considering that the peroxidation of unsaturated fatty acids is a prerequisite for ferroptosis to occur, this result further supports the hypothesis that CML exhibits heightened susceptibility to ferroptosis. These results collectively indicate an aberrant regulation of ferroptosis in CML samples, which may have implications for the initiation and progression of the disease.

### The correlation between the ferroptosis score and the immune microenvironment as well as signaling pathways

**Figure 2:**
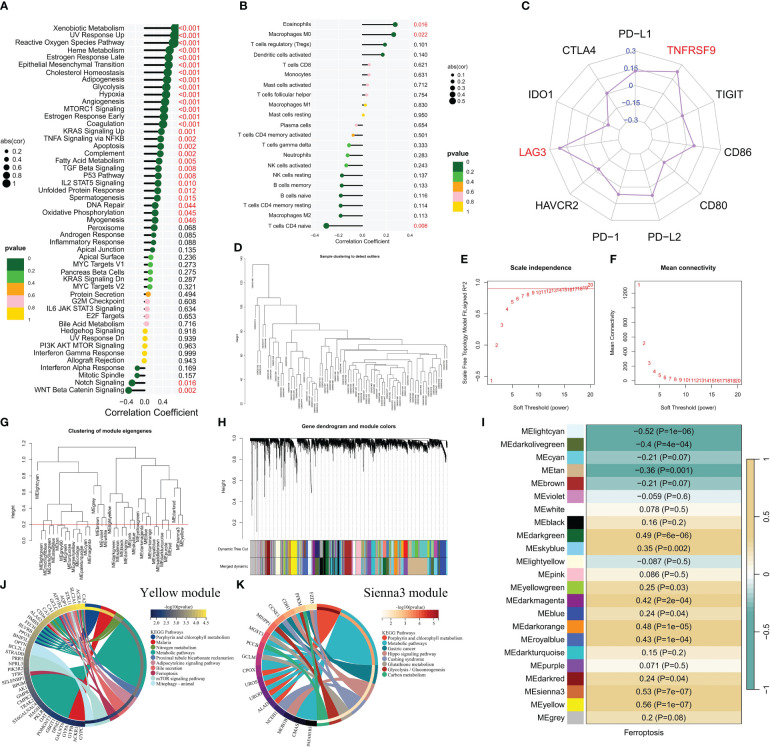
The correlation between the ferroptosis score and the immune microenvironment and signaling pathways. **(A-C)** Correlation analysis revealed associations between the ferroptosis score and enrichment scores of tumor marker gene sets **(A)**, infiltration of immune cells **(B)**, and expression of immune checkpoints **(C)**. **(D)** Cluster plot displaying CML samples. **(E, F)** Scale-free fitting index and average connectivity were used to analyze various soft threshold powers. **(G)** Clustering was performed on different modules, with a cutting height set at 0.2 represented by the red line. **(H)** Cluster plots were generated based on different measures using 1-TOM calculation. **(I)** Heatmap illustrating the correlation between module genes and ferroptosis score. **(J, K)** KEGG enrichment analysis was conducted for yellow module genes, as well as sienna3 module genes.

The relationship between the ferroptosis score and the immune microenvironment of CML as well as cancer pathways was further analyzed. It was observed that there were significant associations between the ferroptosis score and key tumor marker pathways, including xenobiotic metabolism, reactive oxygen species pathway, heme metabolism, and epithelial mesenchymal transition activities (**Figure 2A**). Moreover, positive correlations were found with glycolysis and hypoxia, while negative correlations were observed with Notch signaling and WNT beta-catenin signaling. These findings suggest that an increased activity in the ferroptosis pathway is accompanied by enhanced cancer cell metabolism. Immune infiltration analysis revealed a positive correlation between the ferroptosis score and eosinophil infiltration, M0 macrophage infiltration, as well as regulatory T cell (Treg) infiltration; meanwhile, a negative correlation was identified with naive CD4+ T cells (**Figure 2B**). Furthermore, a significant positive correlation was also found between the ferroptosis score and gene expression of immune checkpoints LAG3 and TNFRSF9 (**Figure 2C**), indicating potential immunosuppression among patients with high ferroptosis scores.

To gain a deeper understanding of the underlying mechanisms associated with ferroptosis in CML, we conducted WGCNA to explore the network of co-expressed genes significantly correlated with ferroptosis scores. The cluster dendrogram depicted the clustering characteristics of all CML samples (**Figure 2D**). **Figures 2E, F** illustrate the scale-free fit exponential and average connectivity analysis for various soft threshold powers. We set the cut height at 0.2 to include modules exhibiting a correlation coefficient greater than 0.8 (**Figure 2G**). Based on an optimal soft threshold power β=15 (unscaled R^2 = 0.9), WGCNA classified the top 5000 genes with the highest standard deviation into 23 independent co-expression modules (**Figure 2H**). The correlograms depicting module-trait relationships revealed that both yellow and sienna3 modules exhibited strong correlations with ferroptosis scores (**Figure 2I**). KEGG enrichment analysis demonstrated that these two modules were enriched in porphyrin and chlorophyll metabolism as well as metabolic pathways (**Figures 2J, K**). Additionally, yellow module genes were found to be associated with nitrogen metabolism, adipocytokine signaling pathway, mTOR signaling pathway, and mitophagy; while sienna3 module genes showed enrichment in hippo signaling pathway, glutathione metabolism, glycolysis/gluconeogenesis, and carbon metabolism. The findings suggest that metabolic reprogramming may contribute to the malignant proliferation of CML cells, while also enhancing the susceptibility of CML cells to ferroptosis by generating higher levels of ROS and unsaturated fatty acids (11, 29).

### Analysis of the diagnostic value of FRG

**Figure 3:**
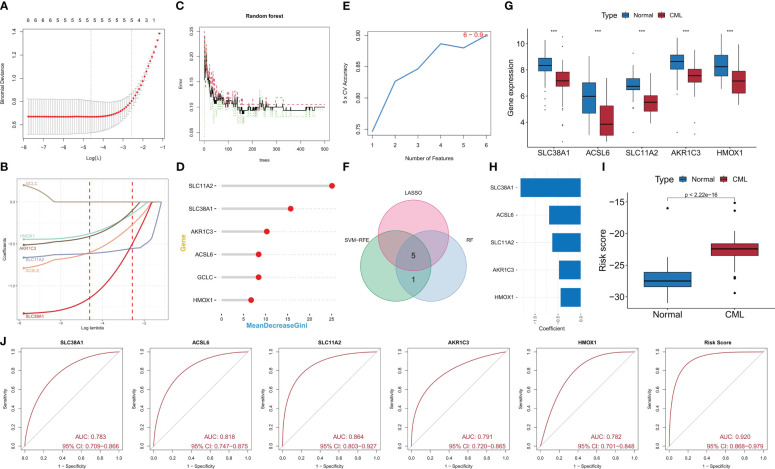
Identification of diagnostic FRG. **(A, B)** Diagnostic FRGs were identified by the LASSO regression algorithm. **(C, D)** Diagnostic FRGs were identified by the RF algorithm. **(E)** Diagnostic FRGs were identified by the SVM-RFE algorithm. **(F)** Venn diagram of variables identified by LASSO, RF, and SVM-RFE algorithms. **(G)** Differences in expression of the three diagnostic FRGs between CML samples and normal samples in the GSE13159 cohort. **(H)** Coefficients of risk score model. **(I)** Differences in risk score between CML samples and normal samples in the GSE13159 cohort. **(J)** ROC curve analysis was used to evaluate the diagnostic value of the five FRGs and risk score in the GSE13159 cohort.

We conducted further analysis on the diagnostic value of FRG in CML. Three machine learning algorithms, namely LASSO, RF, and SVM-RFE, were employed for dimensionality reduction to select the most informative FRGs. From the DEFRGs, we identified 5, 6, and 6 variables that accurately distinguished CML samples from normal samples, respectively (**Figures 3A–E**). Among these variables, there were five overlapping diagnostic FRGs (ACSL6, SLC11A2, HMOX1, SLC38A1, and AKR1C3) included among them (**Figure 3F**). The expression levels of all five FRGs were significantly downregulated in CML samples compared to normal samples (**Figure 3G**). Using LASSO regression analysis, we developed a risk score model to assess the combined diagnostic value of FRG (**Figure 3H**, **Supplementary Table S1**). The risk score levels were significantly elevated in the CML samples (**Figure 3I**). ROC curve analysis revealed high diagnostic AUC values for ACSL6 (0.818), SLC11A2 (0.864), HMOX1 (0.782), SLC38A1(0.783), AKR1C3(0.791), as well as for the risk score (0.920) (**Figure 3J**). The combination of these five FRGs further improved their diagnostic value.

### Validation of the diagnostic value of FRG and analysis of their role in the evaluation of therapeutic effect

**Figure 4:**
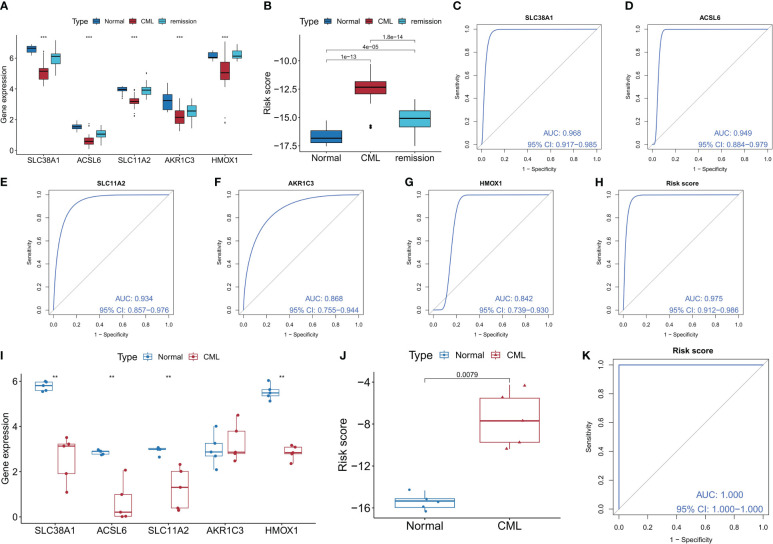
Validation of the diagnostic value of the diagnostic FRG. **(A, B)** Differences in expression of the five diagnostic FRGs and risk score between CML samples and normal samples in the GSE144119 cohort (The Kruskal-Wallis test was employed for the comparison among the three groups). **(C-H)** ROC curve analysis was used to evaluate the diagnostic value of the five FRGs and risk score in the GSE144119 cohort. **(I, J)** Differences in expression of the five diagnostic FRGs and risk score between CML samples and normal samples in our clinical cohort. **(K)** ROC curve analysis was used to evaluate the diagnostic value of risk score in our clinical cohort. **p < 0.01; ***p < 0.001.

We confirmed the diagnostic value of the five FRGS. In the GSE144119 cohort, we observed a significant decrease in expression levels of all five FRGS in CML samples, which showed partial restoration after treatment response (**Figure 4A**). Furthermore, the risk score levels were significantly increased in CML samples and exhibited a significant decrease after treatment remission (**Figure 4B**), thereby demonstrating the therapeutic evaluation value of FRG. ROC curve analysis revealed that ACSL6, SLC11A2, HMOX1, SLC38A1, AKR1C3, and the risk score model had AUC values of 0.949, 0.934, 0.868, 0.842, and 0.975 respectively (**Figures 4C-H**); thus confirming their diagnostic value in CML cases. In our clinically independent cohort study, we also observed a significant decrease in ACSL6, SLC11A2, HMOX1, and SLC38A1 expression in CML samples while AKR1C3 did not show a significant difference due to small sample size issues (**Figure 4I**). The risk score levels were also significantly increased in CML samples (**Figure 4J**). ROC curve analysis demonstrated an AUC value of 1 for the risk score model (**Figure 4K**). Clinical sample-based sequencing data further verified the high diagnostic value associated with these five FRGs in CML. In conclusion, we have identified highly reliable FRGs which could potentially serve as a novel adjunctive tool for clinical diagnosis and treatment decision-making in patients with CML.

### Analysis of the differential diagnostic value of FRG

**Figure 5:**
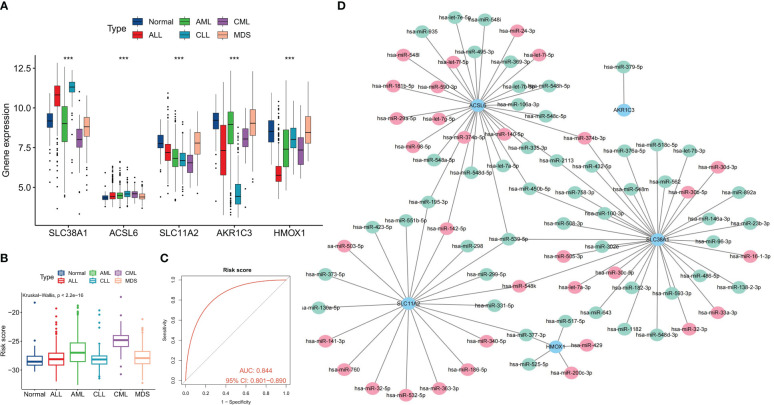
Differential diagnostic value of the five FRGs in CML and other hematological malignancies. **(A)** Expression differences of the five diagnostic FRGs among CML, AML, CLL, ALL, MDS, and normal samples. **(B)** differences in risk scores among CML, AML, CLL, ALL, MDS, and normal samples. **(C)** ROC curve analysis of risk scores in CML and other hematological malignancies. **(D)** Regulatory network of miRNAs and the five diagnostic FRGs; red indicates miRNA expression is up-regulated in CML samples, and green indicates expression is down-regulated. ***p < 0.001.

We conducted a comprehensive analysis to evaluate the differential diagnostic value of the five FRGs. The GSE13159 cohort included sequencing data from 750 ALL samples, 542 AML samples, 448 CLL samples, and 206 MDS samples. Interestingly, the expression levels of most FRGs, including SLC38A1, SLC11A2, and HMOX1, were found to be lower in CML samples compared to other types of hematologic tumors. Conversely, ACSL6 exhibited higher expression levels (**Figure 5A**). Furthermore, subsequent calculations revealed that CML samples displayed the highest risk score (**Figure 5B**). ROC curve analysis demonstrated that the risk score effectively distinguished CML from other hematological malignancies with high accuracy (AUC=0.844) (**Figure 5C**). The diagnostic value of FRG has been systematically evaluated, and we have also endeavored to investigate the regulatory mechanisms governing FRG expression. In this study, our focus lies on miRNA, as we aim to construct a miRNA regulatory network to identify potential miRNAs that could inhibit FRG expression by binding to FRG in CML cells (**Figure 5D**).

### Identification of ferroptosis-related molecular subtypes and analysis of differences in biological characteristics between subtypes

**Figure 6:**
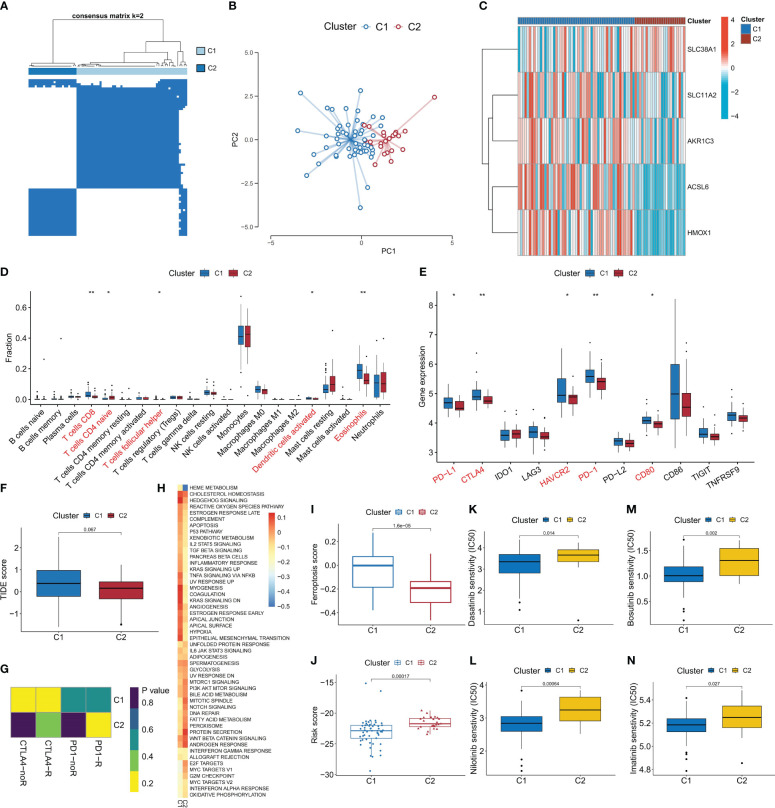
Identification of ferroptosis-related molecular subtypes and analysis of their differences in biological characteristics and treatment sensitivity. **(A)** Based on the expression of DEFRG, CML patients were divided into two ferroptosis-related molecular subtypes by consensus clustering algorithm. **(B)** PCA algorithm was used to analyze the distribution differences of patients between subtypes. **(C-F)** Differences in expression of DEFRG **(C)**, infiltration of 22 immune cells **(D)**, expression of immune checkpoints **(E)**, TIDE score **(F)**, immunotherapy response **(G)**, activity of tumor hallmark gene sets **(H)**, ferroptosis scores **(I)**, risk score **(J)**, and therapeutic sensitivity to four TKIs **(K-N)** between the two molecular subtypes. *p < 0.05; **p < 0.01.

To comprehensively analyze the biological significance of FRGs in CML, we utilized the expression profiles of the five diagnostic FRGs in CML samples to identify two distinct molecular subtypes, namely Cluster C1 and Cluster C2, employing a consensus clustering algorithm (**Figure 6A**, **Supplementary Table S2**). The distribution characteristics of these two molecular subtypes were further confirmed by PCA, revealing significant and discernible differences (**Figure 6B**). Subsequently, through heatmap visualization, it was observed that ACSL6, SLC11A2, HMOX1, and AKR1C3 exhibited up-regulation in subtype C1 while SLC38A1 displayed higher expression levels in subtype C2 (**Figure 6C**). To explore additional distinctions between these subtypes at a biological level, immune infiltration analysis demonstrated that subtype C1 had an increased proportion of CD8+ T cells, follicular helper T cells, activated dendritic T cells, and eosinophils compared to subtype C2 (**Figure 6D**). Furthermore, there were notable variations in the expression levels of immune checkpoint genes; specifically within subtype C1 where PD-L1, CTLA-4, HAVCR2, PD-1, and CD80 showed elevated expressions (**Figure 6E**). This suggests that subtype C1 may exhibit certain immunosuppressive tendencies leading to potential exhaustion of CD8+ T cells. These findings were corroborated by higher TIDE scores for subtype C1 (**Figure 6F**). Conversely, C2subtype appeared more likely to benefit from immunotherapy (**Figure 6G**).

Additionally, our GSVA analysis revealed that the C1 subtype demonstrates heightened activation of signal transduction pathways such as hedgehog signaling and TNFA signaling via NFKB (**Figure 6H**). Moreover, we observed increased activity in cancer-promoting pathways including hypoxia and reactive oxygen species pathway. In contrast, the C2 subtype exhibited elevated activity in proliferation-related pathways such as G2M checkpoint, E2F targets, and MYC targets V1. Notably, C1 displayed a higher ferroptosis score while C2 had a higher risk score (**Figures 6I, J**). Drug prediction analysis indicated that imatinib, nilotinib, dasatinib, and bosutinib demonstrated greater efficacy against subtype C1 compared to subtype C2 (**Figures 6K-N**). These findings will significantly contribute to the development of personalized treatment strategies for patients with CML.

### *In vitro* experiments confirmed that CML-resistant cells were more sensitive to ferroptosis treatment

**Figure 7:**
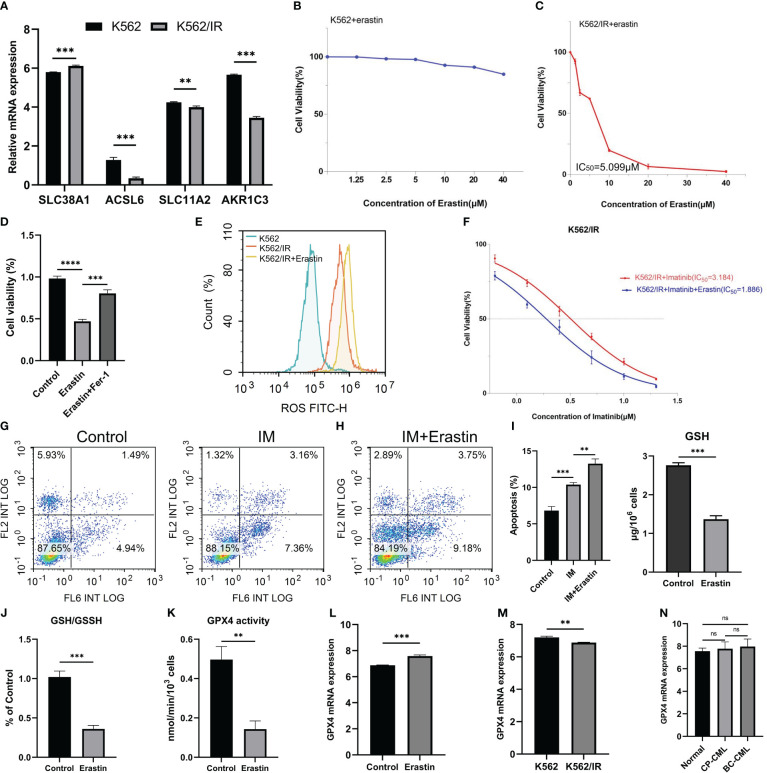
Therapeutic effects of erastin on CML cells. **(A)** Analysis of FRG expression between K562 and K562/IR cells. **(B, C)** Effect of different concentrations of erastin on cell viability of K562 and K562/IR cells after 48h treatment. **(D)** The activity of K562/IR cells after treatment with 5 μM erastin and the addition of 1μM ferroptosis inhibitor Fer-1 for 48h. **(E)** ROS levels in K562, K562/IR, and K562/IR were treated with 5 μM erastin after 24h. **(F)** Changes in cell viability with or without 1.25 μM erastin and treated with different concentrations of imatinib for K562/IR after 48h treatment. **(G, H)** Changes in apoptosis levels after K562/IR treatment with or without 1.25 μM erastin and 1 μM imatinib of 24h. **(I-L)** Changes of GSH level, GSH/GSSH ratio, GPX4 activity, and GPX4 mRNA expression in K562/IR cells after 5 μM erastin treatment for 48h. **(M, N)** The difference in GPX4 mRNA expression between K562 and K562/IR cells, as well as among normal samples, CP-CML samples, and BC-CML samples. The IC50 value of the drug was calculated by GraphPad software. **p < 0.01; ***p < 0.001; ns, no significance.

The expression of five FRGs was detected in CML cell lines K562 and imatinib-resistant cell lines K562/IR. In comparison to K562, SLC38A1 expression showed a slight up-regulation in K562/IR, whereas ACSL6, SLC11A2, and AKR1C3 expressions were down-regulated (HMOX1 gene expression was not detected and therefore not shown) (**Figure 7A**). In our study above, our preliminary analysis indicated that CML cells may exhibit sensitivity to ferroptosis, while CML cells in blast crisis demonstrate resistance towards TKI treatment and potentially higher sensitivity. To validate these findings, we conducted *in vitro* experiments. However, it was observed that the CML cell line K562 did not display sensitivity to erastin-induced ferroptosis (**Figure 7B**); nevertheless, erastin exhibited a certain cytotoxic effect on imatinib-resistant K562 cells (K562/IR) with an IC50 of 5.099 μM (**Figure 7C**). Furthermore, treatment of K562/IR cells with the ferroptosis inhibitor Fer-1 significantly restored cellular viability (**Figure 7D**). Compared to K562 cells, there was a significant increase in ROS levels within K562/IR cells which further escalated after erastin treatment-indicating ROS as a crucial factor for inducing ferroptosis (**Figure 7E**). Additionally, it was discovered that low-dose erastin enhanced the therapeutic sensitivity of imatinib towards K562/IR cells by reducing the IC50 from 3.184 μM to 1.886 μM (**Figure 7F**). Moreover, low-dose erastin promoted apoptosis levels in K562/IR cells treated with imatinib (**Figures 7G, H**). GSH and GPX4 are important indicators of ferroptosis. We found that after erastin treatment, GSH content, GSH/GSSH ratio, and GPX4 enzyme activity of K562/IR cells were significantly decreased, and GPX4 mRNA expression level was slightly increased (**Figures 7I-L**), indicating that erastin inhibited GSH production. In turn, the GPX4 enzyme activity is reduced, which can not inhibit the production of excess ROS, resulting in ferroptosis of K562/IR cells. Finally, we also detected GPX4 expression in K562 and K562/IR cells and CML samples, and the results showed that GPX4 expression in K562/IR cells was lower than that in K562 cells, and there was no significant difference in GPX4 expression between BC-CML and CP-CML samples and normal samples (**Figures 7M, N**). These results suggest that important mechanisms of ferroptosis resistance in CML-resistant cells may not be regulated by GPX4.

## Discussion

Ferroptosis, a newly discovered mode of cell death in recent years, plays a crucial role in regulating various physiological and pathological processes (10). In the context of tumors, ferroptosis is closely associated with the biological characteristics of tumor cells. The hypoxic microenvironment easily triggers the generation of ROS, while the lipid metabolism required for rapid proliferation creates favorable conditions for lipid peroxidation (7). These features collectively indicate that tumor cells are inclined to undergo ferroptosis. The induction of ferroptosis in tumor cells and the attenuation of their protective capacity have significant clinical value for cancer therapy, aiming to enhance tumor cell death or develop novel targeted therapies against apoptosis resistance (30).

In this study, we conducted a systematic analysis of ferroptosis levels in samples from patients with CML using transcriptome sequencing data. Our findings confirm the clinical significance of FRG in diagnosing and evaluating treatment outcomes for CML. Analysis of data from multiple cohorts reveals a significant reduction in ferroptosis scores in CML samples, which further decreases with disease progression. Non-responders also exhibit lower ferroptosis scores compared to CML patients who respond to TKI therapy. Subsequent analyses indicate that lower ferroptosis scores may be associated with decreased expression of genes involved in suppressing ferroptosis, suggesting that CML cells with weaker inhibition against ferroptosis may be more susceptible to induction therapy targeting this process. Through additional cell experiments, we validate that CML-resistant cells are more sensitive to the induction of ferroptosis and can enhance the sensitivity of imatinib treatment, providing a novel target and strategy for overcoming drug resistance in CML. Furthermore, our results demonstrate that the ferroptosis score serves as an informative indicator reflecting the characteristics of the tumor microenvironment in CML. Patients with high ferroptosis scores exhibit increased infiltration by Tregs and higher expression levels of immune checkpoint genes LAG3 and TNFRSF9, which are associated with immunosuppression. Additionally, there is a positive correlation between ferroptosis scores and activity levels within most tumor signature pathways. By conducting WGCNA analysis, we have further identified metabolic pathways as crucial determinants influencing the activity of the ferroptosis pathway itself. Therefore, metabolic reprogramming plays a crucial role not only in promoting malignant proliferation but also contributes to triggering ferroptosis (8, 29).

The expression profile and clinical significance of FRG were further analyzed in this study. The majority of differentially expressed FRGs were found to be down-regulated in CML samples, suggesting their potential involvement in the pathogenesis of CML. Additionally, these FRGs were found to participate in various metabolic pathways, highlighting their multifaceted functions beyond regulating ferroptosis. To comprehensively validate the diagnostic value of FRG, three machine learning algorithms were employed to identify five CML-specific diagnostic FRGs: ACSL6, SLC11A2, HMOX1, SLC38A1, and AKR1C3. These genes showed significantly reduced expression levels in CML samples compared to normal samples.

The diagnostic value of these five FRGs was confirmed not only within the analysis cohort and validation cohort but also in a real-world clinical cohort. This comprehensive validation enhanced the performance of the risk score model based on their expression levels for diagnosing CML patients accurately. Furthermore, it was observed that as treatment remission occurred in CML patients, the expression levels of FRGs increased while the risk scores decreased accordingly. Importantly, these five FRGs can also be utilized for distinguishing CML from other hematological malignancies with clinical relevance. These bioinformatics findings provide strong evidence supporting the diagnostic and therapeutic evaluation potential of FRG specifically in CML patients. Additionally, based on distinct patterns of FRG expressions identified through our analysis approach, we classified two molecular subtypes within the population of CML patients: subtype C1, characterized by a higher proportion of CD8+ T cell infiltration and elevated immune checkpoint gene expressions suggesting immunosuppression; these patients are predicted to exhibit greater sensitivity towards TKI treatments compared to subtype C2. In conclusion, the proposed molecular subtypes will significantly enhance our understanding of the distinct disease characteristics exhibited by patients with CML, thereby providing valuable insights for tailored clinical guidance in personalized treatment strategies.

Finally, we discovered through further experimentation that CML-resistant cells exhibited heightened sensitivity to ferroptosis, potentially due to elevated levels of ROS in these cells. In tumor cells, ROS acts as a signaling molecule and promotes various phenotypes such as growth, metastasis, resistance to apoptosis, and differentiation disorders by activating survival signaling pathways, accelerating energy metabolism, and generating carcinogenic mutations (31). Numerous studies have also confirmed that ROS serves as a major source of genomic instability in different types of cancer. The continuous mutation of cancer cell genomes is a significant cause of drug resistance and relapse in cancer therapy (32, 33). Multiple studies have also substantiated the reasons behind the substantial increase in ROS levels observed in CML-resistant cells. This primarily stems from the activation of various downstream signaling pathways by BCR-ABL1, including the PI3K/AKT/mTOR pathway which enhances glucose metabolism and mitochondrial electron transport chain activity excessively (34, 35); augmentation of NADPH oxidase activity (36); and regulation of target gene transcription for ROS generation via STAT5 (37). Accumulation of ROS drives a cycle of genomic instability leading to BCR-ABL1 mutations or other chromosomal aberrations along with TKI resistance resulting in drug resistance. Additionally, high levels of ROS can induce oxidative damage to mitochondrial DNA within CML-resistant cells causing mitochondrial dysfunction that disrupts the oxidative respiratory chain leading to excessive electron leakage thereby further increasing ROS production within resistant cells (38). Elevated levels of ROS facilitate the formation of more heteromutations while stimulating the signaling capacity within cancer pathways thus generating additional alternative mechanisms promoting CML resistance. Therefore, elevated levels of ROS play a pivotal role in rendering CML-resistant cells more susceptible to ferroptosis, thereby offering a novel therapeutic avenue for overcoming CML resistance. Currently, numerous regulatory mechanisms associated with ferroptosis have been elucidated, including the involvement of HDAC3 via the Hippo signaling pathway (39). Further exploration into the mechanism underlying ferroptosis in CML is warranted.

In summary, we have elucidated the molecular characteristics of ferroptosis in CML from a bioinformatics perspective. The findings from these analyses will contribute to a deeper understanding of the biological significance of ferroptosis in CML. FRG, identified through various machine learning algorithms and validated across multiple cohorts, demonstrates reliable clinical diagnostic value. Moreover, the introduction of ferroptosis-associated molecular subtypes has significantly enhanced our comprehension of individualized traits among CML patients and facilitated personalized treatment strategies. The induction of ferroptosis may also serve as a promising therapeutic approach for overcoming resistance in CML. However, our study does have certain limitations, including the need for a larger sample size to validate the bioinformatics findings, more cell lines and more comprehensive experiments to elucidate the regulatory mechanisms underlying ferroptosis in CML-resistant cells. In subsequent studies, we will expand our sample collection and enhance our exploration of relevant mechanisms through both *in vivo* and *in vitro* experiments.

## Conclusion

The transcriptomic analysis conducted in this study has revealed the molecular characteristics of ferroptosis in samples from patients with CML. By employing machine learning algorithms, reliable clinical diagnostic value was successfully identified for FRG expression patterns. This understanding of individual molecular subtypes associated with ferroptosis can effectively guide clinical treatment strategies for CML patients. Furthermore, targeting and inducing ferroptosis shows great promise as a potential therapeutic approach to address drug-resistant CML.

## Data availability statement

The original contributions presented in the study are included in the article/**Supplementary Material**. Further inquiries can be directed to the corresponding authors.

## Ethics statement

The studies involving humans were approved by Ethics Committee of the Second Affiliated Hospital of Nanchang University. The studies were conducted in accordance with the local legislation and institutional requirements. The participants provided their written informed consent to participate in this study.

## Author contributions

FZ: Data curation, Formal analysis, Funding acquisition, Methodology, Resources, Software, Validation, Visualization, Writing – original draft. XZ: Validation, Visualization, Writing – original draft. ZW: Validation, Visualization, Writing – original draft. XL: Funding acquisition, Validation, Visualization, Writing – original draft. BH: Funding acquisition, Validation, Visualization, Writing – original draft. XW: Conceptualization, Funding acquisition, Project administration, Resources, Supervision, Writing – review & editing. GK: Conceptualization, Funding acquisition, Project administration, Resources, Supervision, Writing – review & editing.

## References

[1] Jabbour E Kantarjian H. Chronic myeloid leukemia: 2022 update on diagnosis, therapy, and monitoring. Am J Hematol. (2022) 97:1236–56. doi: 10.1002/ajh.26642 35751859

[2] Osman AEG Deininger MW. Chronic Myeloid Leukemia: Modern therapies, current challenges and future directions. Blood Rev. (2021) 49:100825. doi: 10.1016/j.blre.2021.100825 33773846 PMC8563059

[3] Poudel G Tolland MG Hughes TP Pagani IS. Mechanisms of resistance and implications for treatment strategies in chronic myeloid leukaemia. Cancers (Basel). (2022) 14. doi: 10.3390/cancers14143300 PMC9317051 35884363

[4] Alves R Gonçalves AC Rutella S Almeida AM De Las Rivas J Trougakos IP. Resistance to tyrosine kinase inhibitors in chronic myeloid leukemia-from molecular mechanisms to clinical relevance. Cancers (Basel). (2021) 13. doi: 10.3390/cancers13194820 PMC8508378 34638304

[5] Rosti G Castagnetti F Gugliotta G Baccarani M. Tyrosine kinase inhibitors in chronic myeloid leukaemia: which, when, for whom? Nat Rev Clin Oncol. (2017) 14:141–54. doi: 10.1038/nrclinonc.2016.139 27752053

[6] Ma L Shan Y Bai R Xue L Eide CA Ou J. A therapeutically targetable mechanism of BCR-ABL-independent imatinib resistance in chronic myeloid leukemia. Sci Trans Med. (2014) 6:252ra121. doi: 10.1126/scitranslmed.3009073 PMC4162097 25186176

[7] Mou Y Wang J Wu J He D Zhang C Duan C. Ferroptosis, a new form of cell death: opportunities and challenges in cancer. J Hematol Oncol. (2019) 12:34. doi: 10.1186/s13045-019-0720-y 30925886 PMC6441206

[8] Stockwell B Friedmann Angeli J Bayir H Bush A Conrad M Dixon S. Ferroptosis: A regulated cell death nexus linking metabolism, redox biology, and disease. Cell. (2017) 171:273–85. doi: 10.1016/j.cell.2017.09.021 PMC5685180 28985560

[9] Dixon S Lemberg K Lamprecht M Skouta R Zaitsev E Gleason C. Ferroptosis: an iron-dependent form of nonapoptotic cell death. Cell. (2012) 149:1060–72. doi: 10.1016/j.cell.2012.03.042 PMC3367386 22632970

[10] Jiang X Stockwell B Conrad M. Ferroptosis: mechanisms, biology and role in disease. Nat Rev Mol Cell Biol. (2021) 22:266–82. doi: 10.1038/s41580-020-00324-8 PMC8142022 33495651

[11] Xie Y Hou W Song X Yu Y Huang J Sun X. Ferroptosis: process and function. Cell Death differentiat. (2016) 23:369–79. doi: 10.1038/cdd.2015.158 PMC5072448 26794443

[12] Yang W SriRamaratnam R Welsch M Shimada K Skouta R Viswanathan V. Regulation of ferroptotic cancer cell death by GPX4. Cell. (2014) 156:317–31. doi: 10.1016/j.cell.2013.12.010 PMC4076414 24439385

[13] Bersuker K Hendricks J Li Z Magtanong L Ford B Tang P. The CoQ oxidoreductase FSP1 acts parallel to GPX4 to inhibit ferroptosis. Nature. (2019) 575:688–92. doi: 10.1038/s41586-019-1705-2 PMC6883167 31634900

[14] Hu Q Wei W Wu D Huang F Li M Li W. Blockade of GCH1/BH4 axis activates ferritinophagy to mitigate the resistance of colorectal cancer to erastin-induced ferroptosis. Front Cell Dev Biol. (2022) 10:810327. doi: 10.3389/fcell.2022.810327 35223839 PMC8866854

[15] Wu J Minikes AM Gao M Bian H Li Y Stockwell BR. Intercellular interaction dictates cancer cell ferroptosis via NF2-YAP signalling. Nature. (2019) 572:402–6. doi: 10.1038/s41586-019-1426-6 PMC6697195 31341276

[16] Lee H Zandkarimi F Zhang Y Meena JK Kim J Zhuang L. Energy-stress-mediated AMPK activation inhibits ferroptosis. Nat Cell Biol. (2020) 22:225–34. doi: 10.1038/s41556-020-0461-8 PMC7008777 32029897

[17] Zou Y Palte MJ Deik AA Li H Eaton JK Wang W. A GPX4-dependent cancer cell state underlies the clear-cell morphology and confers sensitivity to ferroptosis. Nat Commun. (2019) 10:1617. doi: 10.1038/s41467-019-09277-9 30962421 PMC6453886

[18] Yusuf RZ Saez B Sharda A van Gastel N Yu VWC Baryawno N. Aldehyde dehydrogenase 3a2 protects AML cells from oxidative death and the synthetic lethality of ferroptosis inducers. Blood. (2020) 136:1303–16. doi: 10.1182/blood.2019001808 PMC7483435 32458004

[19] Du J Wang T Li Y Zhou Y Wang X Yu X. DHA inhibits proliferation and induces ferroptosis of leukemia cells through autophagy dependent degradation of ferritin. Free Radical Biol Med. (2019) 131:356–69. doi: 10.1016/j.freeradbiomed.2018.12.011 30557609

[20] Zhong FM Yao FY Liu J Zhang HB Zhang J Zhang N. Ferroptosis-related molecular patterns reveal immune escape, inflammatory development and lipid metabolism characteristics of the tumor microenvironment in acute myeloid leukemia. Front Oncol. (2022) 12:888570. doi: 10.3389/fonc.2022.888570 36518303 PMC9742468

[21] Liu S Wu W Chen Q Zheng Z Jiang X Xue Y. TXNRD1: A key regulator involved in the ferroptosis of CML cells induced by cysteine depletion *in vitro* . Oxid Med Cell Longev. (2021) 2021:7674565. doi: 10.1155/2021/7674565 34917232 PMC8670935

[22] Yu G Wang LG Han Y He QY. clusterProfiler: an R package for comparing biological themes among gene clusters. Omics: J Integr Biol. (2012) 16:284–7. doi: 10.1089/omi.2011.0118 PMC3339379 22455463

[23] Hänzelmann S Castelo R Guinney J. GSVA: gene set variation analysis for microarray and RNA-seq data. BMC Bioinf. (2013) 14:7. doi: 10.1186/1471-2105-14-7 PMC3618321 23323831

[24] Newman A Liu C Green M Gentles A Feng W Xu Y. Robust enumeration of cell subsets from tissue expression profiles. Nat Methods. (2015) 12:453–7. doi: 10.1038/nmeth.3337 PMC4739640 25822800

[25] Langfelder P Horvath S. WGCNA: an R package for weighted correlation network analysis. BMC Bioinf. (2008) 9:559. doi: 10.1186/1471-2105-9-559 PMC2631488 19114008

[26] Wilkerson M Hayes D. ConsensusClusterPlus: a class discovery tool with confidence assessments and item tracking. Bioinf (Oxford England). (2010) 26:1572–3. doi: 10.1093/bioinformatics/btq170 PMC2881355 20427518

[27] Geeleher P Cox N Huang R. pRRophetic: an R package for prediction of clinical chemotherapeutic response from tumor gene expression levels. PloS One. (2014) 9:e107468. doi: 10.1371/journal.pone.0107468 25229481 PMC4167990

[28] Li SQ Liu J Zhang J Wang XL Chen D Wang Y. Transcriptome profiling reveals the high incidence of hnRNPA1 exon 8 inclusion in chronic myeloid leukemia. J advanced Res. (2020) 24:301–10. doi: 10.1016/j.jare.2020.04.016 PMC7210475 32405436

[29] Zheng J Conrad M. The metabolic underpinnings of ferroptosis. Cell Metab. (2020) 32:920–37. doi: 10.1016/j.cmet.2020.10.011 33217331

[30] Zhang C Liu X Jin S Chen Y Guo R. Ferroptosis in cancer therapy: a novel approach to reversing drug resistance. Mol Cancer. (2022) 21:47. doi: 10.1186/s12943-022-01530-y 35151318 PMC8840702

[31] Sabharwal SS Schumacker PT. Mitochondrial ROS in cancer: initiators, amplifiers or an Achilles’ heel? Nat Rev Cancer. (2014) 14:709–21. doi: 10.1038/nrc3803 PMC4657553 25342630

[32] Srinivas US Tan BWQ Vellayappan BA Jeyasekharan AD. ROS and the DNA damage response in cancer. Redox Biol. (2019) 25:101084. doi: 10.1016/j.redox.2018.101084 30612957 PMC6859528

[33] Cui Q Wang JQ Assaraf YG Ren L Gupta P Wei L. Modulating ROS to overcome multidrug resistance in cancer. Drug resistance updates: Rev commentaries antimicrobial Anticancer chemother. (2018) 41:1–25. doi: 10.1016/j.drup.2018.11.001 30471641

[34] Antoszewska-Smith J Pawlowska E Blasiak J. Reactive oxygen species in BCR-ABL1-expressing cells - relevance to chronic myeloid leukemia. Acta Biochim Polonica. (2017) 64:1–10. doi: 10.18388/abp.2016_1396 27904889

[35] Kim JH Chu SC Gramlich JL Pride YB Babendreier E Chauhan D. Activation of the PI3K/mTOR pathway by BCR-ABL contributes to increased production of reactive oxygen species. Blood. (2005) 105:1717–23. doi: 10.1182/blood-2004-03-0849 15486067

[36] Reddy MM Fernandes MS Salgia R Levine RL Griffin JD Sattler M. NADPH oxidases regulate cell growth and migration in myeloid cells transformed by oncogenic tyrosine kinases. Leukemia. (2011) 25:281–9. doi: 10.1038/leu.2010.263 PMC4078661 21072051

[37] Warsch W Grundschober E Berger A Gille L Cerny-Reiterer S Tigan AS. STAT5 triggers BCR-ABL1 mutation by mediating ROS production in chronic myeloid leukaemia. Oncotarget. (2012) 3:1669–87. doi: 10.18632/oncotarget.806 PMC3681503 23458731

[38] Glowacki S Synowiec E Blasiak J. The role of mitochondrial DNA damage and repair in the resistance of BCR/ABL-expressing cells to tyrosine kinase inhibitors. Int J Mol Sci. (2013) 14:16348–64. doi: 10.3390/ijms140816348 PMC3759915 23965958

[39] Meng H Yu Y Xie E Wu Q Yin X Zhao B. Hepatic HDAC3 regulates systemic iron homeostasis and ferroptosis via the hippo signaling pathway. Res (Washington D.C.). (2023) 6:281. doi: 10.34133/research.0281 PMC10687581 38034086

